# Analysis of BoDV-1 status, EEG resting-state alpha activity and pro-inflammatory cytokines in adults with and without major depressive disorder

**DOI:** 10.3389/fpsyg.2024.1499446

**Published:** 2024-11-21

**Authors:** Anna J. Torner, Bernhard T. Baune, Kristian Folta-Schoofs, Detlef E. Dietrich

**Affiliations:** ^1^Neurodidactics & NeuroLab, Institute of Psychology, University of Hildesheim, Hildesheim, Germany; ^2^Department of Psychiatry, University of Münster, Münster, Germany; ^3^Department of Psychiatry, Melbourne Medical School, The University of Melbourne, Melbourne, VIC, Australia; ^4^The Florey Institute of Neuroscience and Mental Health, The University of Melbourne, Melbourne, VIC, Australia; ^5^AMEOS Clinical Center Hildesheim, Hildesheim, Germany; ^6^Center for Mental Health, Hannover Medical School, Hannover, Germany; ^7^Center for Systems Neuroscience Hannover, Hannover, Germany

**Keywords:** major depressive disorder, Borna disease virus 1, interleukin-6, interleukin-8, EEG, alpha band

## Abstract

**Introduction:**

In severe cases, an infection with the Borna Disease Virus 1 (BoDV-1), the causative agent of Borna disease in horses, sheep, and other domestic mammals, was reported to be accompanied by cognitive dysfunctions, seizures, deep coma, or severe to fatal encephalitis in humans. In addition, asymptomatic or mild courses of BoDV-1 infection are discussed to act as a co-factor in the etiology of Major Depressive Disorder (MDD). Previously, studies using electroencephalography (EEG) reported BoDV-1-dependent changes in event-related potentials (ERPs), thus indicating the use and added value of non-invasive studies in Borna research.

**Methods:**

Here, we examined possible connections between BoDV-1 status, EEG restingstate alpha activity, and serum levels of pro-inflammatory Interleukin 6 (IL-6) and Interleukin 8 (IL-8) in MDD patients and in a comparison group of adults without MDD diagnosis.

**Results:**

Interestingly, for both groups, we revealed a comparable high number of BoDV-1 positive and BoDV-1 negative participants. Compared to adults without MDD diagnosis, MDD patients showed a decrease in their relative EEG alpha power at posterio-central, but increased values at anterio-central electrode sites. Most important, no group-dependent effect of BoDV-1 status on EEG resting-state activity had been observed. Compared to BoDV-1 positive and negative adults without MDD diagnosis, as well as BoDV-1 positive MDD patients, BoDV-1 negative MDD patients revealed a comparatively weak significant negative correlation between relative fronto-central EEG alpha power and concentrations of pro-inflammatory IL-8.

**Discussion:**

Taken together, our data confirm MDD-dependent alterations in EEG resting-state alpha activity, which, however, were not accompanied by major BoDV-1 dependent neurophysiological or immunological effects. Future – probably more invasive – studies further have to clarify the significance of the observed negative correlation between relative fronto-central EEG alpha power and concentrations of pro-inflammatory IL-8.

## Highlights

- This is the first study examining EEG resting-state alpha activity together with pro-inflammatory cytokines IL-6 and IL-8 in BoDV-1 positive and BoDV-1 negative adults with and without MDD diagnosis.
- Our data revealed a comparable high number of BoDV-1 positive and BoDV-1 negative adult participants with and without MDD diagnosis.
- EEG findings of this study confirm MDD-dependent alterations in EEG resting-state alpha activity, which, however, were not accompanied by major BoDV-1 dependent neurophysiological or immunological effects.
- However, results indicate a weak negative correlation between relative fronto-central EEG alpha power and concentrations of pro-inflammatory IL-8 in the group of BoDV-1 negative MDD patients. Further, probably more invasive, studies have to clarify the significance of the observed negative correlation.

## 1 Introduction

Borna Disease Virus 1 (BoDV-1; species Mammalian orthobornavirus 1) is a non-cytolytic negative single-stranded RNA-virus that belongs to the zoonotic members of the family Bornaviridae (Bode et al., 1996; Bode et al., 2022; Dietrich et al., 2020; Hornig et al., 2003; Lieb and Staeheli, 2001; Ludwig and Bode, 2009; Rubbenstroth et al., 2021). BoDV-1 is known as the causative agent of Borna disease in horses, sheep, and other domestic mammals, and is maintained in bicolored white-toothed shrews (*Crocidura leucodon*). The virus spreads from nasal mucous membranes via axonal and transsynaptic pathways into the limbic system (including hippocampus, amygdala, and hypothalamus) of the brain, where it can persist and might gain influence on the glutamate system and the kainite KA1 receptor, as well as on the release of pro-and anti-inflammatory cytokines (Carbone et al., 2001; Dietrich, 2011; Dietrich and Bode, 2008; Dietrich et al., 1998, 2000, 2020; Gosztonyi, 2008; Gosztonyi et al., 2020; Marty et al., 2022). Although there is yet no clear indication of any human-to-human transmission, it has been shown that living in rural or suburban environments, direct or indirect contact to animals preying on infected shrews (e.g., by cats), or agricultural work might facilitate BoDV-1 transmission to humans (Niller et al., 2020).

In humans, symptomatic, oligosymptomatic, and asymptomatic courses of BoDV-1 infection had been assumed (Coras et al., 2019; Dietrich et al., 2020; Eisermann et al., 2021; Finck et al., 2020; Frank et al., 2022; Korn et al., 2018; Liesche et al., 2019; Ludwig and Bode, 2009; Niller et al., 2020; Schlottau et al., 2018). Acute and severe infections were reported to promote peripheral neuropathy, seizures, deep coma or severe to fatal encephalitis (Frank et al., 2022; Niller et al., 2020; Schlottau et al., 2018; Tappe et al., 2019). Non-severe clinical symptoms of BoDV-1 infection comprise headache and fever, as well as a general feeling of illness, reduced vigilance, and mild to moderate cognitive impairments. In humans, BoDV-1-specific diagnostic procedures differ with respect to their detection methods, and are not regularly indicated during the acute phase of infection (Frank et al., 2022). Thus, epidemiology and clinical outcomes differ and are still discussed controversially (Bode et al., 2020). Ludwig and Bode (2009) suggested a BoDV-1 dependent seroprevalence of 30% for asymptomatic infected humans, and of 5% for manifested BoDV-1 cases in the general population, but a recent seroprevalence study demonstrated only a low rate of BoDV-1 infection (0.14%) in German veterinarians (who represent a possible risk group for infection), and no cases of infection were reported for blood donors (Tappe et al., 2019). For the first time, Nakamura et al. (2000) documented the detection of BoDV-1 RNA and antigen in the brain of a BoDV-1 seropositive 24-years old schizophrenic patient (undifferentiated type) developing depression, anxiety, and general fatigue. For this patient, a histopathological examination revealed mild inflammatory changes predominantly within the brain’s hippocampus (Nakamura et al., 2000). Consistent with these findings, BoDV-1 infection markers, including BoDV-1-specific circulating immune complexes (CICs) and plasma antigen (pAG), were regularly reported in psychiatric patients with Major Depressive Disorder (MDD; Mazaheri-Tehrani et al., 2014; Wang et al., 2014). Recent clinical support for a possible role of BoDV-1 in the etiopathology of depression comes from a recent study, demonstrating MDD patients to benefit from an antiviral treatment against BoDV-1 using amantadine (Dietrich et al., 2020).

Compared to healthy people, MDD patients reveal alterations at the cognitive, affective, motivational and/or somatic level, comprising symptoms of cognitive impairment, low levels of self-esteem, depressed mood, feelings of guilt or hopelessness, as well as a loss of interest, a decrease in motivation, and/or sleep disturbances (American Psychiatric Association, 2013; Berking and Radkovsky, 2012; de Jong-Meyer, 2011; Rock et al., 2014; World Health Organization, 2016). BoDV-1-specific CICs and pAG were shown to correlate with both the severity of depressive symptoms in MDD (Bode et al., 2001; Dietrich et al., 2020; Mazaheri-Tehrani et al., 2014), and alterations in limbic and frontal brain regions (Dietrich et al., 2005; Zhang et al., 2022). Up to now, two electroencephalography (EEG) studies were able to observe BoDV-1-dependent changes in event-related potentials (ERPs). Using a Go/NoGo task in patients with Obsessive-Compulsive Disorder (OCD), Dietrich et al. (2005) reported an increase of the N1 component in patients with a high degree of BoDV-1 specific CICs. Similarly, Zhang et al. (2022) investigated OCD patients in a continuous word recognition paradigm and found the old/new effect to be impaired in patients showing higher BoDV-1 specific CICs. Although both studies only employed small sample sizes and thus have to be interpreted with caution, these ERP results suggest that BoDV-1 infection may be associated with attention-related (Dietrich et al., 2005) and memory-related (Zhang et al., 2022) cognitive changes that can be measured using non-invasive electrophysiological methods.

A further promising electrophysiological alternative to ERP studies might be the measurement of changes in EEG resting-state activity, especially when testing MDD patients. Previous studies in MDD patients reported an increase of (frontal) EEG activity in the alpha frequency band, which is indicative of a frontal hypoactivation in the brain of depressive patients (Baxter, 1989; Debener et al., 2000; Fingelkurts and Fingelkurts, 2015; Jaworska et al., 2012; Olbrich and Arns, 2013; Pollock and Schneider, 1990). In addition, hemispheric asymmetries in frontal EEG resting-state alpha activity have been frequently reported in depressive patients and are associated to changes in individuals’ approach-or withdrawal-behavior (Coan and Allen, 2004; Debener et al., 2000; Fingelkurts and Fingelkurts, 2015; Henriques and Davidson, 1991; Jaworska et al., 2012; Knott et al., 2001).

Beside from neurophysiological findings, several studies support the notion of interconnections between MDD and the release of pro-inflammatory cytokines, especially of the chemotactic Interleukin-8 (IL-8) and Interleukin-6 (IL-6), but results are yet inconsistent (Himmerich et al., 2019; Köhler et al., 2017; Tsai, 2021). Pro-inflammatory cytokines promote inflammatory processes, and are thought to negatively affect processes of neurogenesis and the regulation of neurotransmitters. They might gain influence on the limbic system and the hypothalamic–pituitary–adrenal (HPA) axis (Capuron and Miller, 2011; Himmerich et al., 2019; McAfoose and Baune, 2009; Miller et al., 2009; Stuart et al., 2015), which is dysregulated in MDD patients (Berking and Radkovsky, 2012; de Jong-Meyer, 2011). IL-8 is mainly released by microglia, acts as an inflammatory mediator (by enabling neutrophils to migrate to inflammatory sites), and is assumed to be associated with neuroprotective mechanisms (Baggiolini and Clark-Lewis, 1992; Ehrlich et al., 1998; Janelidze et al., 2015; Stuart et al., 2015). Whereas increased IL-8 concentrations were observed in depressive participants (Simon et al., 2008) and in patients with acute or remitted MDD (Birur et al., 2017; Vogelzangs et al., 2016), reduced IL-8 concentrations and a negative correlation between the severity of depression and concentrations of IL-8 (with a higher severity of depression associated to lower IL-8 levels) were reported by Zou et al. (2018). In addition, several studies were unable to reveal significant group differences or correlations (i.e., Dowlati et al., 2010; Eyre et al., 2016; Köhler et al., 2017). Beside from IL-8, IL-6 is considered to be strongly associated with the severity of depressive symptoms (Miller et al., 2009), and this association has also been confirmed for the general population, albeit to a lesser extent (Howren et al., 2009). Compared to healthy controls, several studies reported increased IL-6 levels in MDD patients (Dowlati et al., 2010; Goldsmith et al., 2016; Howren et al., 2009; Köhler et al., 2017), but Zou et al. (2018) found no significant differences in IL-6 concentrations between MDD patients and healthy controls. It was argued that IL-6 changes might be observable only in depressive individuals, suffering from an atypical, energy-related symptom profile, including somatic symptoms (Lamers et al., 2020).

As depression is worldwide one of the most common mental disorders and significantly contributes to the overall global burden of disease (Chesney et al., 2014; World Health Organization, 2023), it is crucial to examine potential factors of its etiopathology, including inflammatory processes (e.g., induced by viral infection), which have increasingly been investigated in recent decades (Capuron and Miller, 2011; Himmerich et al., 2019; Miller et al., 2009). Moreover, a higher incidence of (zoonotic) viruses constitutes a major health threat to humans (Craemer, 2023; Meyding-Lamadé and Craemer, 2023). Although previous studies suggested both BoDV-1 and pro-inflammatory cytokines IL-6 and IL-8 as possible co-factors in the etiopathology of depression, currently, little is known about cognitive-affective, immunological, as well as neurophysiological changes associated with BoDV-1 infection in humans (Dietrich and Bode, 2008; Dietrich et al., 2005). Preliminary investigations indicate an association between BoDV-1 infection markers and BoDV-1-specific changes in ERPs (Dietrich et al., 2005; Zhang et al., 2022), which is of clinical relevance, since EEG methods (in contrast to more invasive measurement techniques) can easily be applied in diagnostic settings. However, further studies, employing large sample sizes, are required to confirm the previously reported results. In addition, EEG studies examining BoDV-1-specific changes in an EEG resting-state condition might be promising, because (hemispheric) changes in the EEG alpha frequency range were regularly observed in depressive patients (Baxter, 1989; Coan and Allen, 2004; Debener et al., 2000; Fingelkurts and Fingelkurts, 2015; Henriques and Davidson, 1991; Jaworska et al., 2012; Knott et al., 2001; Olbrich and Arns, 2013; Pollock and Schneider, 1990). Hence, the objective of our study was to examine potential associations between the status of BoDV-1 infection, concentrations in pro-inflammatory cytokines IL-6 and IL-8, as well as amplitudes in EEG resting-state alpha activity in both MDD patients and non-depressive healthy adults.

## 2 Materials and methods

### 2.1 Participants

Our study was performed in a total of *N* = 66 participants that were assigned to a group of MDD patients and a group of adults without MDD diagnosis. The MDD group contained a total of *N* = 38 patients (23 females, 15 males, *Mean*_age_ = 40.82 years ±2.09 *S.E*., Age-range: 19 to 65 years) with a medical diagnosis of MDD, comprising a current or previous major depressive episode in accordance to DSM-IV-TR (American Psychiatric Association, 2000) criteria. Only patients without any previous diagnosis or high screening score of a psychotic disorder, eating disorder, learning disorder, autistic spectrum disorder, or any other medical or physical conditions, affecting the central nervous system (e.g., multiple sclerosis, Parkinson’s disease, dementia) were included. Data from *N* = 35 MDD patients were collected as part of the Cognitive Function and Mood Study (CoFaM-Study; Baune and Air, 2016), and were tested within the Burghof Clinic (Rinteln, Germany). Additional data from *N* = 3 MDD patients were acquired in the AMEOS Clinical Center (Hildesheim, Germany). From the MDD group, a total of *N* = 4 patients had to be excluded due to poor quality of EEG recordings (e.g., lack of artifact-free intervals or less than 65% of critical segments remained after artifact correction), resulting in *N* = 34 MDD patients (20 females, 14 males, *Mean*_age_ = 41.62 years ±2.19 *S.E*., Age-range: 21 to 65 years), which remained for further analyses.

The comparison group contained a total of *N* = 28 adults without a diagnosis of MDD (all females, *Mean*_age_ = 21.29 years ±0.57 *S.E*., Age-range: 19 to 34 years). These participants were tested at the University of Hildesheim (Hildesheim, Germany).

Before signing an informed consent form, all participants were provided with a detailed explanation (written and in person) of study procedures and measures. The study was conducted according to the guidelines of the Declaration of Helsinki, and was approved by the Human Research Ethics Committee at the University of Adelaide (Approval Number: H-160-2011), the Royal Adelaide Hospital Human Research Ethics (Approval Number: 111230), and the ethics committee of the FB-1 (Educational and Social Sciences) at University of Hildesheim (Reference Number: 223/2022).

### 2.2 Plasma analyses

For the analyses of serum/plasma levels of biomarkers, samples of peripheral blood were collected always between 8:30 a.m. and 11:30 a.m. (within less than 3 min to avoid the initiation of pituitary stress responses; Vahl et al., 2005). Blood samples were centrifuged (1800–2,122 × g, 10–15 min) and frozen at least at −20° in a refrigerator (monitored by a central alert system 24 h/day and 7 days/week). Using an enzyme immune assay (EIA; Dedimed GmbH Europarc Lab, Kleinmachnow, Germany, MVZ DIAMEDIS, Sennestadt, Germany), BoDV-1-specific CICs, generally considered to reflect a positive (i.e., an activated) status of BoDV-1 infection, were detected (using a cut-off value of 0.1). Using flow cytometry and the BD Cytometric Bead Array (CBA) Human Inflammatory Cytokines Kit (Becton, Dickinson and Company BD Biosciences, San Jose, USA), lithium heparin plasma from MDD patients of the Burghof Clinic was analyzed according to concentrations of pro-inflammatory cytokines IL-6 and IL-8. The analysis was conducted by the Lab Division of Molecular Neurobiology of Mental Health (Münster, Germany). For participants tested in Hildesheim, concentrations of IL-6 and IL-8 were detected from their serum using a chemiluminescent immune assay (CLIA; using an IL-8 detection limit of 5.00 pg/ml and an IL-6 detection limit of 3.50 pg/ml). This analysis was conducted by the MVZ Laboratory Limbach (Lehrte, Germany).

### 2.3 EEG recordings and EEG analysis

Resting-state EEG activity (with eyes closed) was continuously recorded from passive (GVB-geliMED GmbH, Bad Segeberg, Germany; EEG recordings in Rinteln) or active AgCl electrodes (actiCAP; Brain Products GmbH, Gilching, Germany; EEG recordings in Hildesheim) using a NeuroPrax (NeuroConn, Ilmenau, Germany; EEG recordings in Rinteln) or BrainAmp DC amplifier (Brain Products GmbH, Gilching, Germany; EEG recordings in Hildesheim). Dependent on the amplifier, sampling rate was either 512 or 1,000 Hz (down-sampled to 512 Hz). Brain electrical activity was recorded from electrode positions Fp1, Fp2, F7, F3, Fz, F4, F8, T3, C3, Cz, C4, T4, T5, P3, Pz, P4, T6, O1, and O2 (EEG recordings in Hildesheim additionally employed FC5, FC1, FC2, FC6, CP5, CP1, CP2, CP6, and Oz). All electrode positions were defined according to the international 10–20 system. Electrode impedances were kept below 5 kΩ for passive electrodes, and below 20 kΩ for active electrodes. As reference, linked ears (A1, A2; EEG recordings in Rinteln), or FCz (re-referenced to mastoid positions TP9 and TP10; EEG recordings in Hildesheim) were used. AFz was used as ground. For EEG recordings conducted in Rinteln, no electrooculographic (EOG) activity was recorded, but EEG recordings in Hildesheim employed two additional EOG electrodes, which were placed below and near the outer canthus of the right eye. EEG resting-state recordings were conducted in a semi-darkened dim and quiet room. All participants were seated comfortably on a chair with neck support. EEG-activity was recorded for a total duration of 10 min (EEG recordings in Hildesheim) or 20 min (EEG recordings in Rinteln). All participants were informed to close their eyes and to remain relaxed, yet alert. During the recording, participants from the Burghof Clinic were additionally instructed to open their eyes for a short duration of 5 s after 3, 6, 15 and 18 min of recording, to hyperventilate after 10 min of recording (for a total duration of 2 min), and to relax afterwards. However, these sections of the EEG were not analyzed for this study.

All EEG raw data were analyzed offline using the BrainVision Analyzer 2.0 (Brain Products, Gilching, Germany). After down-sampling and re-referencing, EEG data were bandpass filtered using a low cut-off value (order 8) of 0.1 Hz (Mohammadi et al., 2015), and a high cut-off value (order 4) of 40 Hz (Neuner et al., 2014). In addition, a 50 Hz-Notch filter had been applied. Using manual raw data inspection, EEG data were visually inspected to mark artifacts and identify artifact-free intervals for the further analysis. Due to missing EOG channels in recordings from the Burghof Clinic, all ocular artifacts were detected using an Independent Component Analysis (ICA). Signal components, which were topographically associated to ocular activity, were subtracted from the EEG signal (Neuner et al., 2014). Afterwards, an artifact-free interval of at least 2 min (placed between 1 and 8 min of EEG recording) was selected for further analysis. EEG data from the chosen interval were segmented into 2-s epochs (50% overlap) and DC-and baseline-corrected, before artifacts with amplitudes over ±75μV (related to muscle tension, tremor, or drowsiness) were marked as bad intervals (Mohammadi et al., 2015). Participants with less than 65% of remaining artifact-free segments were excluded from further analysis. The mean proportion of segments remaining for the further analysis was 91.50% ± 1.05 *S.E*. (range: 65 to 99%). A Fast Fourier Transformation (FFT; symmetric Hanning window of 5%; according to Mohammadi et al., 2015) was employed to extract relative power values (i.e., absolute values per 0.5 Hz bin normalized with respect to the frequency range of 0.5 to 34 Hz). FFT-transformed segments (using a bin-width of 0.5 Hz) were averaged for each channel, before individual frequency band values were exported. In accordance to both the guideline of the International Federation of Clinical Neuropsychology (Nuwer et al., 1999) and Neuner et al. (2014), frequency bands were defined as followed: delta (0.5–3.5 Hz), theta (4–7 Hz), alpha (7.5–12 Hz), and beta (13–34 Hz). For detailed statistical analyses, mean relative power values were extracted for the alpha band at fronto-central (Fz), fronto-medial (F3 and F4), centro-central (Cz), centro-medial (C3 and C4), as well as for parieto-central (Pz) and parieto-medial (P3 and P4) electrode positions. In addition, a spatially averaged value across 19 electrodes, which were employed for all EEG recordings, was computed for each participant.

### 2.4 Statistical analyses

All statistical analyses were conducted using the Statistical Package for the Social Sciences (SPSS; Version 28 & 29 for Mac, IBM, New York, USA). Descriptive statistics were calculated and presented as mean and standard error (*S.E.*). Pearson’s Chi-Square (*χ^2^*) tests were used to examine categorical data. For comparative analyses, repeated measurement analysis of variances (*ANOVAs*) with *post-hoc* (Bonferroni-corrected) pairwise comparisons were employed. Where appropriate, Greenhouse–Geisser corrected *p*-values were applied. Pearson’s correlation coefficient *r* was used to determine possible correlations between variables. For all analyses, the significance level was set to *p* < 0.05.

## 3 Results

### 3.1 Status of BoDV-1 infection

Based on the analyses of blood samples, MDD patients and participants from the comparison group were divided into subgroups of BoDV-1 positive and BoDV-1 negative participants. In the clinical group, *N* = 14 MDD patients (41.2%; 9 females, 5 males, *Mean*_age_ = 40.14 years ±3.90 *S.E*., Age-range: 21 to 58 years, 12 participants on medication) were identified as BoDV-1 positive (indicating an activated status of BoDV-1 infection), whereas *N* = 20 patients (58.8%; 11 females, 9 males, *Mean*_age_ = 42.65 years ±2.59 *S.E.*, Age-range: 23 to 65 years, 15 participants on medication) were tested as BoDV-1 negative (indicating no BoDV-1 infection or a non-activated status of BoDV-1 infection). In the group of adults without MDD diagnosis, *N* = 14 participants (50%; all females, *Mean*_age_ = 20.00 years ±0.30 *S.E*., Age-range: 19 to 23 years) were identified as BoDV-1 positive, whereas *N* = 14 participants (50%; all females, *Mean*_age_ = 22.57 years ±1.00 *S.E.*, Age-range: 19 to 34 years) were tested as BoDV-1 negative. Most important, no significant association between the status of BoDV-1 infection (positive vs. negative) and the clinical diagnosis of MDD (yes vs. no) could be observed (*p* > 0.05). Regarding to sex, no differences could be observed within each study group for BoDV-1 positive vs. BoDV-1 negative subgroups (*p* > 0.05). However, comparisons between MDD patients vs. adults without MDD diagnosis revealed a significant sex difference for BoDV-1 positive subgroups [*χ^2^*(1) = 6.09, *p* < 0.05, *Cramer’s ν* = 0.47] and BoDV-1 negative subgroups [*χ^2^*(1) = 8.57, *p* < 0.01, *Cramer’s ν* = 0.50]. Regarding to age, between-groups comparisons revealed BoDV-1 positive participants without MDD diagnosis to differ from BoDV-1 negative participants without MDD diagnosis [*t*(26) = 2.47, *p* < 0.05, *Cohen’s d* = 0.93]. Such an age difference was not observed for clinical subgroups (*p* > 0.05). In addition, comparisons between MDD patients vs. adults without MDD diagnosis revealed a significant age difference for BoDV-1 positive subgroups [*t*(26) = 5.16, *p* < 0.001, *Cohen’s d* = 1.95] and BoDV-1 negative subgroups [*t*(32) = 6.24, *p* < 0.001, *Cohen’s d* = 2.18]. Finally, clinical subgroups did not differ with respect to their proportion of participants using psychotropic drugs (*p* > 0.05).

### 3.2 Resting-state EEG (with eyes-closed) and mean relative EEG alpha power

**Figure 1:**
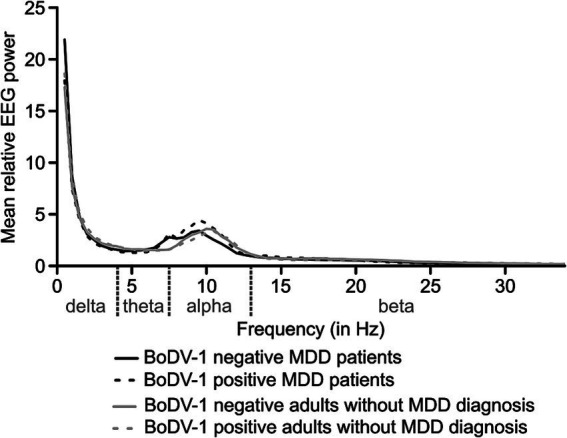
The means of spatially averaged values of mean relative EEG power (for the complete frequency range from 0.5 to 34 Hz; EEG frequency bands are indicated in light gray) for subgroups of patients with Major Depressive Disorder (MDD; dark gray) and BoDV-1 negative (i.e., no active BoDV-1 infection; *N* = 20) or BoDV-1 positive infection status (i.e., active BoDV-1 infection; *N* = 14, dashed line), as well as adults without MDD diagnosis (light gray) and BoDV-1 negative (*N* = 14) or BoDV-1 positive infection status (*N* = 14, dashed line). BoDV-1, Borna Disease Virus; EEG, Electroencephalogram; Hz, Hertz.

For all subgroups, the means of spatially averaged values of relative EEG power (including all EEG frequencies from 0.5 to 34 Hz) are summarized in Figure 1. As it was expected for recordings of resting-state EEG in a closed-eyes condition, a significant increase in power of the EEG alpha frequency band (7.5–12 Hz) was observed for all study groups.

**Figure 2:**
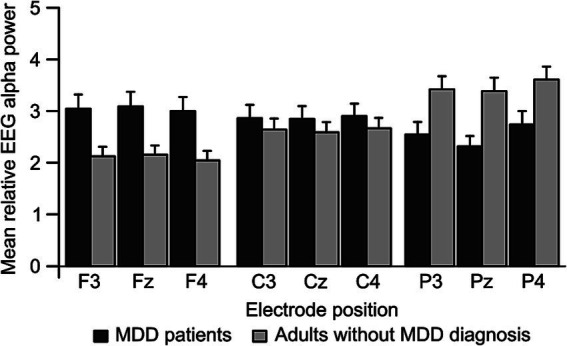
Mean EEG alpha power as it was observed for frontal, central and parietal electrode positions of the left (F3, C3, and P3) and right (F4, C4, and P4) hemisphere in patients with Major Depressive Disorder (MDD; *N* = 38, dark gray) and adults without MDD diagnosis (*N* = 28, light gray). Error bars represent standard error. EEG, Electroencephalography; MDD, Major Depressive Disorder.

To further test for relative power-differences in the EEG alpha band, a 2 × 2 × 2 × 3 repeated measurement *ANOVA* with the between-subjects factors “Group” (MDD patients vs. adults without MDD diagnosis) and “Status of BoDV-1 infection” (positive vs. negative), as well as the within-subjects factors “Hemisphere” (left vs. right hemispheric electrodes) and “Electrode position” (frontal, central, and parietal) was performed. The analysis revealed a significant main effect of “Electrode position” [*F*(1.40, 81.48) = 17.05, *p* < 0.001, *η^2^_p_* = 0.23], as well as a significant interaction between the factors “Group” and “Electrode position” [*F*(1.40, 81.48) = 45.65, *p* < 0.001, *η^2^_p_* = 0.44; see Figure 2]. All remaining main effects and interactions proved to be non-significant (*p* > 0.05). As this *ANOVA* did not reveal any hemisphere-specific effects, a 2 × 2 × 3 repeated measurement *ANOVA* was computed considering only midline “electrode positions” (Fz, Cz, and Pz) as a within-subjects factor, and “Group” (MDD patients vs. adults without MDD diagnosis) as well as “Status of BoDV-1 infection” (positive vs. negative) as between-subjects factors. This analysis confirmed a significant interaction between the factors “Group” and “Electrode position” [*F*(1.36, 79.08) = 39.10, *p* < 0.001, *η^2^_p_* = 0.40; see Figure 2]. All remaining interactions and main effects proved to be non-significant (*p* > 0.05).

For MDD patients in comparison to adults without MDD diagnosis, *post-hoc* comparisons showed a significant increase in mean relative EEG alpha power at midline electrode positions Fz (*Mean*_MDD_ = 3.115 ± 0.241 *S.E*., *Mean*_without MDD_ = 2.155 ± 0.262 *S.E.*, |*Mean*|_Diff_ = 0.959, *p* < 0.01), as well as a significant decrease at electrode position Pz (*Mean*_MDD_ = 2.380 ± 0.219 *S.E.*, *Mean*_without MDD_ = 3.385 ± 0.238 *S.E.,* |*Mean*|_Diff_ = 1.005, *p* < 0.01), but no mean relative EEG alpha power difference at Cz (*Mean*_MDD_ = 2.873 ± 0.228 *S.E.*, *Mean*_without MDD_ = 2.588 ± 0.247 *S.E.,* |*Mean*|_Diff_ = 0.285, *p* > 0.05). More precisely, adults without MDD diagnosis showed a significantly decreased mean relative EEG alpha power at electrode position Fz in comparison to Cz (|*Mean*|_Diff_ = 0.432, *p* < 0.001) and Pz (|*Mean*|_Diff_ = 1.230, *p* < 0.001), as well as a significant decrease at electrode position Cz compared to Pz (|*Mean*|_Diff_ = 0.797, *p* < 0.001). In contrast, MDD patients showed a reversed activation pattern with a significantly increased mean relative EEG alpha power at electrode position Fz in comparison to Cz (|*Mean*|_Diff_ = 0.242, *p* < 0.05) and Pz (|*Mean*|_Diff_ = 0.735, *p* < 0.001), as well as a significant increase at electrode position Cz compared to Pz (|*Mean*|_Diff_ = 0.493, *p* < 0.01).

To summarize, a posterior/parietal to anterior/frontal decrease in mean relative EEG alpha power was observed in adults without MDD diagnosis, whereas a reversed pattern with an increase in EEG alpha power from posterior/parietal to anterior/frontal electrode positions was observed in MDD patients. With respect to BoDV-1 status or hemispheric differences, no significant differences in EEG resting-state alpha activity could be observed.

### 3.3 Concentrations of pro-inflammatory interleukin-6 and interleukin-8

Because of detection limits, IL-8 concentrations <5.00 pg/ml were coded as 4.99 pg/ml, and IL-6 values <3.50 pg/ml were coded as 3.49 pg/ml in all participants. To examine possible differences between concentrations of pro-inflammatory IL-6 or IL-8 with respect to MDD diagnosis and BoDV-1 status, two separate 2 × 2 *ANOVAs* with the factors “Group” (MDD patients vs. adults without MDD diagnosis) and “Status of BoDV-1 infection” (positive vs. negative) were conducted. For mean concentrations of IL-6, the analysis revealed no significant main effects, and no interaction (*p* > 0.05; for descriptive data see Table 1). However, for mean concentrations of IL-8, the analysis revealed a weak, but significant “Group” and “Status of BoDV-1 infection” interaction [*F*(1, 58) = 5.97, *p* < 0.05, *η^2^_p_* = 0.09], with higher IL-8 concentrations in BoDV-1 negative MDD patients compared to BoDV-1 positive MDD patients (|*Mean*|_Diff_ = 3.34, *p* < 0.01) or BoDV-1 negative adults without MDD diagnosis (|*Mean*|_Diff_ = 3.43, *p* < 0.01; see Table 1). However, no difference was observed between BoDV-1 positive and negative participants within the subgroup of adults without MDD diagnosis (|*Mean*|_Diff_ = 1.15, *p* > 0.05), as well as between MDD patients and adults without MDD diagnosis within the subgroup of BoDV-1 positive participants (|*Mean*|_Diff_ = 1.06, *p* > 0.05).

**Table 1:** Summary of descriptive data for measurements of concentrations of pro-inflammatory Interleukin-6 (IL-6) and Interleukin-8 (IL-8), as well as fronto-central relative EEG alpha power in both BoDV-1 negative and BoDV-1 positive MDD patients and adults without MDD diagnosis.

	MDD patients (*N* = 34)		Adults without MDD diagnosis (*N* = 28)	
	BoDV-1 positive (*N* = 14)	BoDV-1 negative (*N* = 20)^1^	BoDV-1 positive (*N* = 14)	BoDV-1 negative (*N* = 14)
IL-6 (pg/ml)				
Mean ± S.E.	4.04 ± 0.30	5.10 ± 0.98	3.49 ± 0.00	3.59 ± 0.10
Range (min - max)	3.49–6.98	3.49–21.20	3.49–3.49	3.49–4.86
IL-8 (pg/ml)				
Mean ± S.E.	7.40 ± 0.53	10.74 ± 1.04	8.46 ± 1.14	7.31 ± 0.47
Range (min-max)	4.99–12.46	4.99–20.94	4.99–21.20	4.99–9.60
Mean relative EEG alpha power in Fz				
Mean ± S.E.	3.25 ± 0.49	2.98 ± 0.34	2.14 ± 0.24	2.17 ± 0.27
Range (min-max)	0.52–7.06	1.09–6.20	0.89–3.49	1.25–4.58

^1^*N* = 19 for IL-6 due to one outlier (>500 pg/ml). BoDV-1, Borna Disease Virus; MDD, Major Depressive Disorder; max, maximum; min, minimum; ml, milliliter; pg, picogram; S.E., standard error.

**Figure 3:**
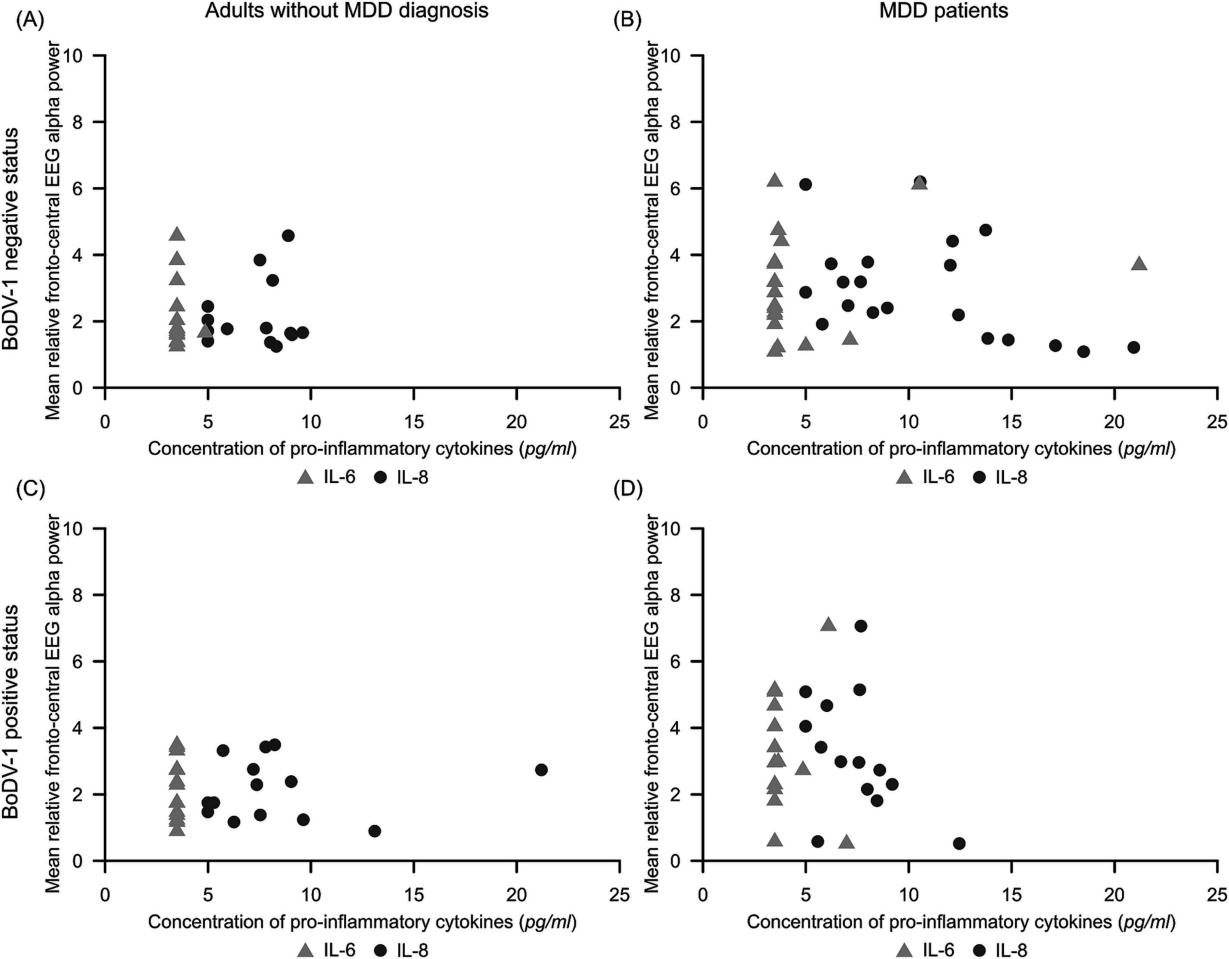
Scatterplots illustrating the variability of mean relative fronto-central EEG alpha power dependent on concentrations of pro-inflammatory IL-6 (light gray triangle) and IL-8 (dark gray circle) for subgroups of (A) BoDV-1 negative adults without MDD diagnosis (*N* = 14), (B) BoDV-1 negative MDD patients (*N* = 20), (C) BoDV-1 positive adults without MDD diagnosis (*N* = 14), and (D) BoDV-1 positive MDD patients (*N* = 14). BoDV-1, Borna Disease Virus; EEG, Electroencephalography; IL-6, interleukin-6; IL-8, interleukin-8; MDD, Major Depressive Disorder; *ml*, milliliter; *pg*, picogram.

Figure 3 illustrates the variability of mean relative fronto-central EEG alpha power in dependence on concentrations of IL-6 and IL-8 for the different subgroups of our study. For participants without MDD diagnosis, IL-6 concentrations were almost always observed below or near the detection limit (*min* to *max* = 3.49 to 4.86 pg/ml; see Table 1). With respect to IL-8 concentrations, subgroups of adults without MDD diagnosis showed IL-8 values to predominately vary between 4.99 and 10.00 pg/ml. In general, concentrations of pro-inflammatory cytokines in participants without MDD diagnosis were – irrespectively of their BoDV-1 status – accompanied by rather low values (<5%) and low variability in fronto-central EEG alpha power (see Figure 3 and Table 1).

The observed result pattern was quite different in MDD patients. Comparable to adults without MDD diagnosis, both subgroups of MDD patients revealed the majority of IL-6 concentrations to lie below or near the detection limit, but demonstrated a slightly higher variability in mean relative fronto-central EEG alpha power (see Figure 3 and Table 1). In BoDV-1 positive MDD patients, the majority of IL-8 concentrations were observed to vary – comparable to IL-8 concentrations in the comparison group – between 4.99 and 10.00 pg/ml, but mean relative fronto-central EEG alpha power showed a high variability of data with relative power values of up to 7% (see Figure 3 and Table 1). However, in the group of BoDV-1 negative MDD patients, participants showed an increased variability of IL-8 concentrations with values ranging from 4.99 to 21.20 pg/ml, showing a concomitant high variability in mean relative fronto-central EEG alpha power with values of up to 6% (Figure 3 and Table 1). Correlation analyses confirmed these observations and revealed a weak, but significant negative correlation between mean IL-8 concentrations and mean relative fronto-central EEG alpha power for BoDV-1 negative MDD patients (*r* = −0.46, *p* < 0.05), and no significant correlations for any of the other study groups (*p* > 0.05).

## 4 Discussion

It is still an open question, if cognitive-affective, behavioral, neurophysiological and/or immunological alterations in MDD patients may be associated to BoDV-1-dependent modulations in limbic and cortical brain regions. The aim of this study was to investigate possible connections between the status of BoDV-1 infection, immunological changes (serum levels of pro-inflammatory IL-6 and IL-8), as well as neurophysiological modulations in closed-eyes EEG resting-state alpha activity in MDD patients and in healthy adults without MDD diagnosis.

For both study groups, we found a comparable number of BoDV-1 positive and BoDV-1 negative participants. In addition, compared to adults without MDD diagnosis, MDD patients showed an increase in anterior/frontal and a decrease in posterior/parietal EEG resting-state alpha power. However, no specific neurophysiological effects of an activated BoDV-1 infection (BoDV-1 positive status) could be observed during closed-eyes EEG resting-state. Interestingly, BoDV-1 negative, but not BoDV-1 positive MDD patients, showed slightly increased concentrations of pro-inflammatory IL-8, which was negatively associated with fronto-central EEG resting-state alpha power.

### 4.1 Proportion of participants with a BoDV-1 positive and BoDV-1 negative status of infection

We observed a similar proportion of participants with a BoDV-1 positive and BoDV-1 negative status of infection (determined through BoDV-1 specific CICs). Differing sensitivities in detection methods impair the comparability of prevalence data reported in different studies (Frank et al., 2022). However, among all methods, BoDV-1 specific CICs, as they were determined in our study, belong to the most frequently used markers of BoDV-1 virus infection, and are considered to reasonably explain infection profiles of dormant and activated states (Bode et al., 2001; Liu et al., 2015). Contrary to previously published studies conducted before the pandemic coronavirus disease 2019 (COVID-19), our results were unable to confirm that MDD patients are overall more likely affected by BoDV-1 infection than healthy individuals without any psychiatric diagnosis (Dietrich et al., 1998; Ludwig and Bode, 2009; Mazaheri-Tehrani et al., 2014; Wang et al., 2014). Our study was conducted during and shortly after the COVID-19 pandemic (i.e., data of adults without MDD diagnosis were assessed shortly after the pandemic, whereas data of most MDD patients, with the exception of two individuals, were collected before the onset of the COVID-19 pandemic). A possible activation of the BoDV-1 infection by the pandemic might explain, why we observed rates of 41.2% for BoDV-1 positive MDD patients and of 50% for adults without MDD diagnosis, which are much higher than many previously reported estimations showing a rather low prevalence of BoDV-1 positive cases, especially for activated states (Ludwig and Bode, 2009; Niller et al., 2020). However, the observed high rate of CICs in BoDV-1 positive MDD patients of our study fits well to previous studies, reporting higher detection rates of BoDV-1 specific RNA transcripts in lymphocytes, increased serum antibody titers against BoDV-1 specific nucleoprotein (p40) and phosphoprotein (p24), and higher BoDV-1 specific CICs in MDD patients (Bode et al., 1995, 2001; Ikuta et al., 2002; Miranda et al., 2006; Sauder et al., 1996). For example, Mazaheri-Tehrani et al. (2014) investigated diagnosed psychiatric patients and reported a high infection (CIC) prevalence of 40.4%, which perfectly matched to our results in MDD patients. On the other hand, for healthy controls, Mazaheri-Tehrani et al. (2014) reported a prevalence of 29.5%, which was rather lower than what we observed for adults without MDD diagnosis. Liu et al. (2015) addressed the risk of BoDV-1 infection in health care settings in China, and found 21.8% of the hospital staff (with the highest prevalence among psychiatry and oncology personnel) to be CIC positive, which exceeded both the prevalence of 18.2% detected in MDD outpatients, and of 11.1% detected in healthy controls. Finally, Bode et al. (2020) reported a prevalence of 57.9% for BoDV-1 infected psychiatric patients in Germany, but again only of 24% for healthy carriers. Interestingly, these authors also reported a prevalence of 37.3% for healthy carriers in the Czech Republic. To summarize, previous studies confirmed a high BoDV-1 infection (CIC) prevalence in psychiatric patients that we also confirmed in our study. However, our study revealed a prevalence of BoDV-1 infection of 50% for healthy carriers, which is much higher than the prevalence rates between 10 and 40%, which were reported for healthy individuals in most of the previous studies. The high prevalence rate observed in our study might reflect an effect of infection with the severe acute respiratory syndrome coronavirus type 2 (SARS-CoV-2) and of COVID-19 on the activation of BoDV-1 infections in healthy carriers. In addition, our observed prevalence rate in healthy participants argues against zoonotic reservoirs as important for the subclinical spread of the BoDV-1 virus (Bode et al., 2020, 2022; Cain and Ly, 2023; Lipkin et al., 2001). Epidemiological surveys are required to further clarify the role of COVID-19 on BoDV-1 infection in humans.

### 4.2 Topographical changes in EEG resting-state alpha activity

Previously, non-invasive EEG studies using ERPs suggest BoDV-1 infection to be associated with neurophysiological and behavioral changes (Dietrich, 2011; Dietrich and Bode, 2008; Dietrich et al., 1998, 2000, 2005, 2020; Gosztonyi, 2008; Gosztonyi et al., 2020; Marty et al., 2022; Zhang et al., 2022). However, in our study, focusing on closed-eyes EEG resting-state alpha activity, we were unable to observe any BoDV-1-dependent changes, which are probably more likely to occur in eyes-opened conditions, where the sensory systems actively process environmental stimuli. In our study, we decided to record EEG activity in an eyes-closed task-free condition to avoid any confounding effects of external stimuli, instructions, or task execution. In addition, resting state seems more self-relevant than standard cognitive tasks and drive subjects to direct their attention away from the (bottom-up) processing of the environment, thus permitting (top-down) processing of the internal mental context (Fingelkurts and Fingelkurts, 2015). The EEG alpha oscillation is commonly thought to arise primarily from the occipital and parietal parts of the cortex (where usually the highest alpha power can be observed), and reflects primarily cortical dendritic activity synchronized across a large part of the visual cortex (Kellaway, 2003; Liu et al., 2012; Tatum et al., 2006; Zou et al., 2009). Furthermore, the thalamus and thalamocortical neurons have been proposed to play an active role in generating and modulating posterior alpha rhythmic activity (Liu et al., 2012). Simultaneously recorded functional magnetic resonance imaging (fMRI) and EEG measurements conducted during rest revealed a negative correlation between posterior alpha-EEG and pulvinar BOLD-fMRI, and a positive correlation between posterior alpha-EEG and anterior as well as medial dorsal (thalamic) nuclei, which appear functionally connected to the cingulate cortex (Liu et al., 2012), forming a cingulo-insular-thalamic network, comprising the dorsal anterior cingulate cortex, anterior insula, anterior prefrontal cortex, and the thalamus (Sadaghiani et al., 2010). This network is proposed (I) to mediate cortical arousal (Saper et al., 2005), (II) to maintain tonic internalized alertness (Coste and Kleinschmidt, 2016; Sadaghiani et al., 2010), (III) to correlate with processes of introspection, internal mentation, or integration of stimulus-independent thought processes (Bowman et al., 2017), and (IV) to correlate with fluctuations in involuntary attention (Dosenbach et al., 2008; Portas et al., 1998). Liu et al. (2012) speculated that while the pulvinar may be intimately involved in the generation of the alpha rhythm in the occipital cortex, such rhythmic activities may be under central modulatory control (Steriade, 1993) effectuated through the anterior and medial dorsal parts of the thalamus. Thus, an increase in posterior alpha (PA) power during rest suggests an (probably primarily pulvinar-mediated) inhibition of external awareness of the environment and a suppression of potentially distracting information (Foxe and Snyder, 2011), whereas a lower PA power may indicate a higher level of cortical arousal and outward vigilance, which may be primarily under central modulatory control of anterior and medial dorsal nuclei (Hopman et al., 2020; Kirschfeld, 2005; Liu et al., 2012). In line with this notion, simultaneously recorded fMRI and EEG measurements conducted during rest showed PA power to be positively correlated with activity in neural regions associated with introspection and rumination (Bowman et al., 2017), and negatively correlated with regions associated with vigilance and visual processing (Laufs et al., 2003). Directing visuospatial attention to a given visual field consistently results in a power suppression of alpha activity over parieto-occipital areas contralateral to the attended visual field (Arana et al., 2022; Händel et al., 2011), showing that modulations of EEG alpha activity correlate with the direction of visuospatial attention. A reduction in the EEG alpha power over parieto-occipital areas had also been reported in children with Attention Deficit Hyperactivity Disorder (ADHD) and frontocortical hypoactivity (Bozhilova et al., 2022; Lenartowicz et al., 2018).

As it was expected from previous studies, we observed a posterior to anterior decrease in EEG alpha power, and – compared to MDD patients – a higher PA power in healthy adults without MDD diagnosis. These data are indicative of a predominance of processes of stimulus-independent and spontaneous self-referential or introspective thought processes in healthy participants in the eyes-closed resting state condition (Bowman et al., 2017). Compared to healthy participants, PA power was significantly reduced in MDD patients, while EEG resting-state alpha activity became significantly increased at anterior/frontal electrode sites (i.e., a reversed topographic activity pattern, compared to adults without MDD diagnosis). This decrease in PA power and increase in anterior alpha (AA) power (representing a frontal hypoactivation; Baxter, 1989; Jaworska et al., 2012) might be indicative of an increased (bottom-up driven) external awareness and outward vigilance in MDD patients during rest, accompanied by a decrease in interoceptive awareness and (top-down driven) impairments to regulate and integrate self-referential processes (Liu et al., 2012; Veer et al., 2010). In line with this notion, decreases in resting-state functional connectivity of the anterior insula (and the related network) have been reported for medication-free MDD patients without comorbidity (Veer et al., 2010).

Beside from modulations in PA power, several clinical studies reported an increase in the asymmetry of AA power (indicative of a left anterior hypoactivation) in acute depressed or remitted psychiatric patients, which was associated with an underactivation of the behavioral approach system, and a bias towards negative-valenced stimuli and an activation of the withdrawal-related behavioral system (Coan and Allen, 2004; Debener et al., 2000; Henriques and Davidson, 1991; Jaworska et al., 2012; Knott et al., 2001; Olbrich and Arns, 2013; Pollock and Schneider, 1990). However, our results were not able to confirm any hemispheric asymmetries in resting-state alpha activity, either for frontal, central or parietal electrode positions.

### 4.3 Pro-inflammatory cytokines in BoDV-1 positive and BoDV-1 negative participants

With regard to the analysis of pro-inflammatory cytokines IL-6 and IL-8, our data are in line with overall inconclusive findings regarding concentrations and effects of pro-inflammatory cytokines in depression (Himmerich et al., 2019; Köhler et al., 2017; Lamers et al., 2020; Tsai, 2021). Contrary to previous findings (Dowlati et al., 2010; Himmerich et al., 2019; Howren et al., 2009; Köhler et al., 2017), our results do not support the assumption of an immune activation in either individuals with or without MDD diagnosis. Most important, the observed concentrations of IL-6 and IL-8 in BoDV-1 positive participants were highly comparable to those observed in BoDV-1 negative adults without MDD diagnosis. Compared to adults without MDD diagnosis, MDD patients failed to demonstrate elevated levels of IL-6, which were previously reported to be associated with the severity of depression (Miller et al., 2009). In our sample (compared to healthy participants), IL-8 was only slightly elevated in BoDV-1 negative, but not in BoDV-1 positive MDD patients. Previous studies (e.g., Hannestad et al., 2011; Iwata et al., 2013) showed that levels of proinflammatory cytokines can be downregulated by psychotropic drugs (e.g., antidepressants), but in our study, BoDV-1 negative and positive MDD patients received comparable medical treatment. Furthermore, serum levels of proinflammatory cytokines could be influenced by demographic factors such as age (Himmerich et al., 2019; Janelidze et al., 2015) and sex (Birur et al., 2017; Kruse et al., 2021). However, in our study, BoDV-1 positive and negative MDD patients did neither differ in age nor sex. One explanation for slightly elevated IL-8 concentrations in BoDV-1 negative, but not in BoDV-1 positive MDD patients might be, that an activation of BoDV-1 in positive MDD patients may bypass the activation of the innate immune system and the release of pro-inflammatory and anti-inflammatory cytokines (Jordan and Lipkin, 2001; Reuter et al., 2010; Zhai et al., 2013). Previous studies have shown that IL-8 might affect neurons and their excitability including the modulation of neurotransmitter release or spontaneous synaptic activity (Giovannelli et al., 1998; Puma et al., 2001). Although the observed negative correlation between IL-8 levels and fronto-central EEG resting-state alpha activity is consistent with the notion that IL-8 may exert neuromodulatory effects (Rostène et al., 2007; Stuart et al., 2015), this finding still needs to be interpreted with caution, and has to be confirmed in future, more systemic studies. These studies should employ larger sample sizes and a combination of non-invasive EEG and invasive measurements, including consideration of a wide range of immunological parameters, endocrine variables, and bioenergetic changes in the intracellular network dynamics of neuron’s mitochondria (Hitzler et al., 2019; Karabatsiakis and Schönfeldt-Lecuona, 2020). The use of invasive methods highlights the overall organic character of both BoDV-1 infections and MDD-related somatic vulnerability, which physiologically affects more than neurons in the brain. For example, BoDV-1-specific changes in the EEG of MDD patients depend on complex changes in inflammatory and hormonal processes that may alter the mitochondrial adenosine triphosphate (ATP) production, which is the main driver of cellular activity, including neuronal activity (Karabatsiakis et al., 2014; Boeck et al., 2018). Invasive measurements may facilitate the identification of potentially important correlations with biological variables outside the central nervous system (CNS), which help to elucidate the precise biological mechanisms leading to observable changes in non-invasive EEG measurements.

## 5 Limitations and conclusion

Our study has several limitations. First, this study was conducted in multiple laboratory settings using different EEG systems, which might have influenced our data. Future studies should ensure the use of consistent experimental procedures with regard to blood analyses and EEG recordings. Second, although the identification of BoDV-1 status based on CICs (using an EIA) is considered a robust method (Bode et al., 2001; Mazaheri-Tehrani et al., 2014), there is currently no standardized procedure available for testing BoDV-1 infection (Bundesministerium für Gesundheit, 2019). Because of an assumed low avidity of human reactive antibodies for BoDV-1 antigens, there is an ongoing controversial discussion about reliable and valid detection methods (Allmang et al., 2001; Lieb and Staeheli, 2001; Schwemmle, 2001). Third, the comparison group of adults without MDD diagnosis only comprised of young female participants, which led to group differences with respect to gender and age in comparison to MDD patients. In addition, the comparison group was tested after the onset of the COVID-19 pandemic, why one cannot exclude that pandemic-related (stressful) conditions or possible infections with SARS-CoV-2 might had an impact on our results. However, a strength of our study was the inclusion of a comparison group, which was – similarly to MDD patients – also tested according to the status of BoDV-1 infection. Overall, it should be emphasized that the observed associations between pro-inflammatory cytokines, resting-state EEG changes and BoDV-1 status in MDD patients should be interpreted with caution. Further studies, using more diverse and a mixture of non-invasive and invasive methods, are needed to confirm and further elucidate the observed correlation. In addition, behavioral testing should be coherently assessed in future studies (Dietrich and Bode, 2008).

To conclude, the present study revealed a comparable number of BoDV-1 positive and BoDV-1 negative participants, possibly due to an activation of BoDV-1 by the COVID-19 pandemic. BoDV-1 status was not shown to exert any effect on EEG resting-state alpha activity. However, MDD patients showed a decreased EEG alpha power at posterior electrode sites, and an increased alpha power at anterior electrode positions, compared to adults without MDD diagnosis. In addition, BoDV-1 negative MDD patients showed slightly increased concentrations of IL-8, which negatively correlated with fronto-central EEG alpha power, supporting previously reported neuromodulating effects of IL-8. Given the high prevalence of MDD, future research should further explore possible modulatory effects of an activated BoDV-1 infection on inflammatory processes and resting-state EEG alpha activity by investigating a larger sample size and employing combined measurements of invasive and non-invasive methods.

## Data Availability

The raw data supporting the conclusions of this article will be made available by the authors, without undue reservation.
